# Luteal Phase Support Using Subcutaneous Progesterone: A Systematic Review

**DOI:** 10.3389/frph.2021.634813

**Published:** 2021-08-06

**Authors:** Alessandro Conforti, Luigi Carbone, Giuseppe Gabriele Iorio, Federica Cariati, Francesca Bagnulo, Vincenzo Marrone, Ida Strina, Carlo Alviggi

**Affiliations:** ^1^Department of Neuroscience, Reproductive Sciences and Odontostomatology, University of Naples Federico II, Naples, Italy; ^2^CEINGE-Biotecnologie Avanzate Scarl, Naples, Italy; ^3^IVF Unit, AOU Policlinico Federico II, Naples, Italy

**Keywords:** progesterone, subcutaneous progesterone, assisted reproductive technology, *in-vitro* fertilization, luteal phase support, luteal phase defect, ovulation induction, systematic review

## Abstract

Luteal phase support (LPS) is crucial in assisted reproductive technology (ART) cycles when the luteal phase has been found to be defective. Such deficiency is most likely related to the supraphysiological steroid levels that usually occurr in stimulated cycles which, in turn, could severely affect luteinizing hormone (LH) secretion and function, thereby negatively influencing the luteal phase. A number of different medications and routes have been successfully used for LPS in ART. Although an optimal protocol has not yet been identified, the existing plethora of medications offer the opportunity to personalize LPS according to individual needs. Subcutaneous administration progesterone has been proposed for LPS and could represent an alternative to a vaginal and intramuscular route. The aim of the present systematic review is to summarize the evidence found in the literature concerning the application of subcutaneous progesterone in ARTs, highlighting the benefits and limits of this novel strategy. With this aim in mind, we carried out systematic research in the Medline, ISI Web of Knowledge, and Embase databases from their inception through to November 2020. Randomized controlled trials (RCTs) were preferred by the authors in the elaboration of this article, although case-control and cohort studies have also been considered. According to our findings, evidence exists which supports that, in women with a good prognosis undergoing a fresh *in vitro* fertilization (IVF) cycle, subcutaneous Pg is not inferior to vaginal products. In the Frozen-thawed embryo transfer (FET) cycle, data concerning efficacy is mixed with an increased miscarriage rate in women undergoing a subcutaneous route in oocyte donor recipients. Data concerning the acceptance of the subcutaneous route versus the vaginal route are encouraging despite the different scales and questionnaires which were used. In addition, a cost-effective analysis has not yet been conducted.

## Introduction

The importance of luteal phase support (LPS) is widely recognized in assisted reproductive technology (ART) and an adequate luteal phase is mandatory for embryo implantation. Under physiological conditions, the luteal phase is mainly sustained by a luteinizing hormone (LH) and a follicle-stimulating hormone (FSH) surge during ovulation, which induces the “luteinization” of the granulosa cells, thereby promoting the formation of the corpus luteum (1). In particular, LH is important, not only during the late phase of folliculogenesis (2–6) and for the support of the corpus luteum (7), but also in the early stage of embryo implantation (8–10). During LP, the corpus luteum sustains the endometrium, preparing it for implantation that usually occurs 6 days after fertilization (2). In medically assisted reproduction, a luteal phase defect is observed. Indeed, the supraphysiological production of sexual steroids during ovarian stimulation (OS) significantly suppresses LH levels, thereby causing impairment of LPS (11). Clinically, the latest Cochrane meta-analysis which included 94 randomized controlled trials (RCTs) (26,198 infertile women undergoing ART) confirmed that LPS is associated with significantly higher live births and ongoing pregnancy compared with placebo or no treatment (12). LPS is carried out mainly with progesterone (Pg) and/or other hormones that promote its production, such as human chorionic gonadotropin (hCG) and the gonadotropin-releasing hormone agonist (GnRHa). Pg supplementation is by far the most common strategy adopted worldwide (11, 13). Pg can be administrated vaginally, rectally, intramuscularly, and orally (12). Oral Pg seems to be more effective, using synthetic Pg rather than natural micronized Pg (11, 14, 15). Notably, among oral products, dydrogesterone seems to offer better results in terms of live births and pregnancy rates in fresh cycles compared with vaginal micronized Pg (16); however, vaginal administration of Pg is still the most preferred route among clinicians (17, 18). Conversely, <6% of practitioners opted for an intramuscular or oral route (13, 17).

Recently, a water-soluble formulation of Pg for subcutaneous administration has been introduced (19, 20). A preliminary study demonstrated that this route can induce endometrial decidualization and present bioavailability similar to oil-based products (19–21). In the present review, we summarize the clinical studies that have adopted subcutaneous Pg for LPS in an ART setting.

## Materials and Methods

### Search Strategy

A systematic search was undertaken in Medline, ISI Web of Knowledge, and EMBASE databases from their inception through to November 2020. Specifically, studies in which subcutaneous Pg was adopted for LPS in women undergoing *in vitro* fertilization (IVF) or intrauterine inseminations (IUI) were selected. RCTs were preferred by the authors in the elaboration of this search, although case-control and cohort studies have been also considered. The following keywords were adopted: LPS, ART, IVF, and assisted reproduction.

### Bias Evaluation

Three authors (AC, VM, and FC) independently evaluated the risk of bias. Senior authors solved conflicts (CA and IS). Eligible RCTs were assessed using the Cochrane risk of bias assessment tool (12, 22). The following issues were assessed in detail: (1) random sequence generation; (2) allocation concealment; (3) binding of participants and personnel; (4) incomplete outcome data; and (5) selective reporting. The risk of bias was graded per consideration as low, unclear, or high.

Non-RCTs were assessed using the Newcastle–Ottawa scale (NOS) (23) score according to three issues: selection of study group, comparability between groups, and exposure/outcome.

## Results

**Figure 1:**
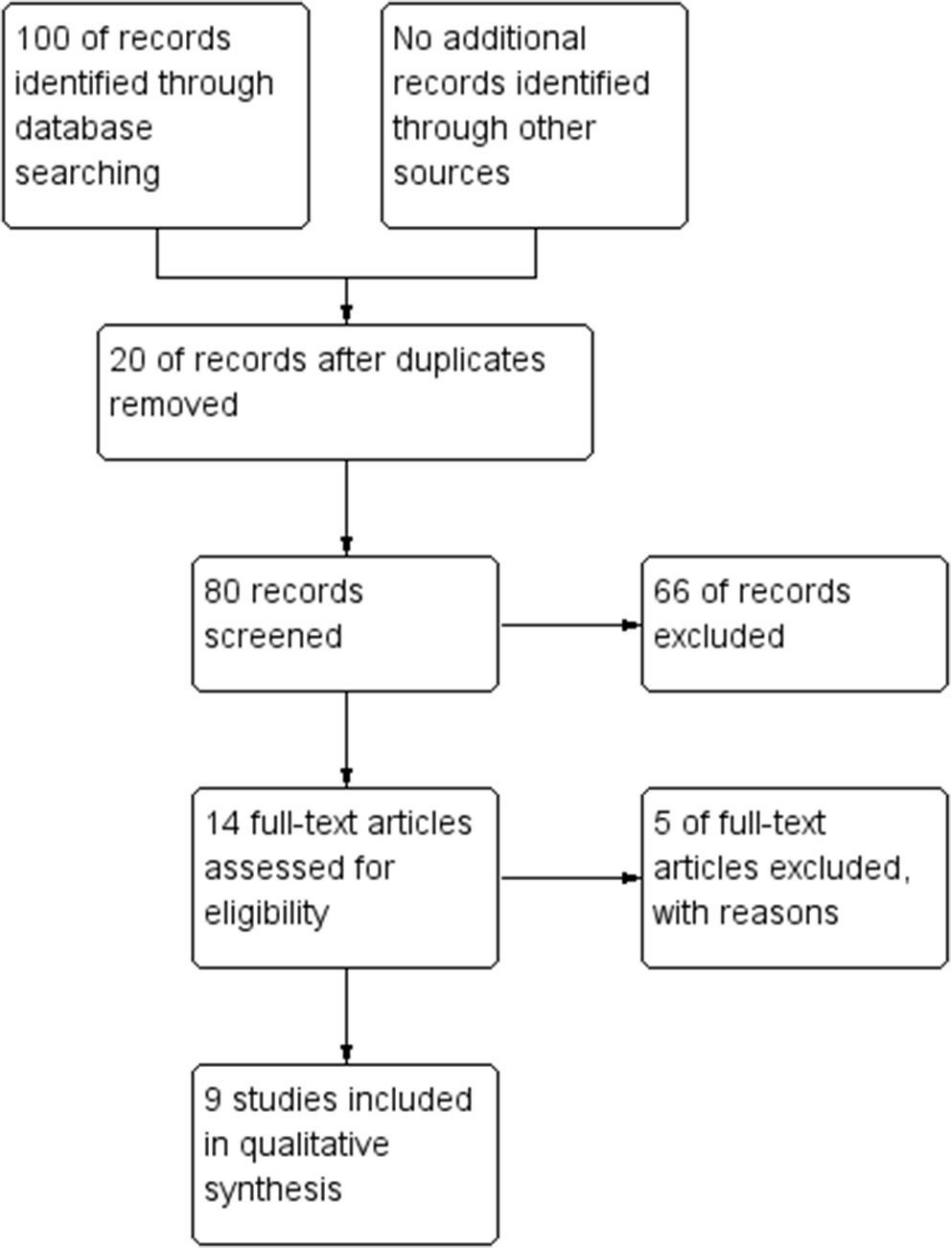
Flow chart.

**Figure 2:**
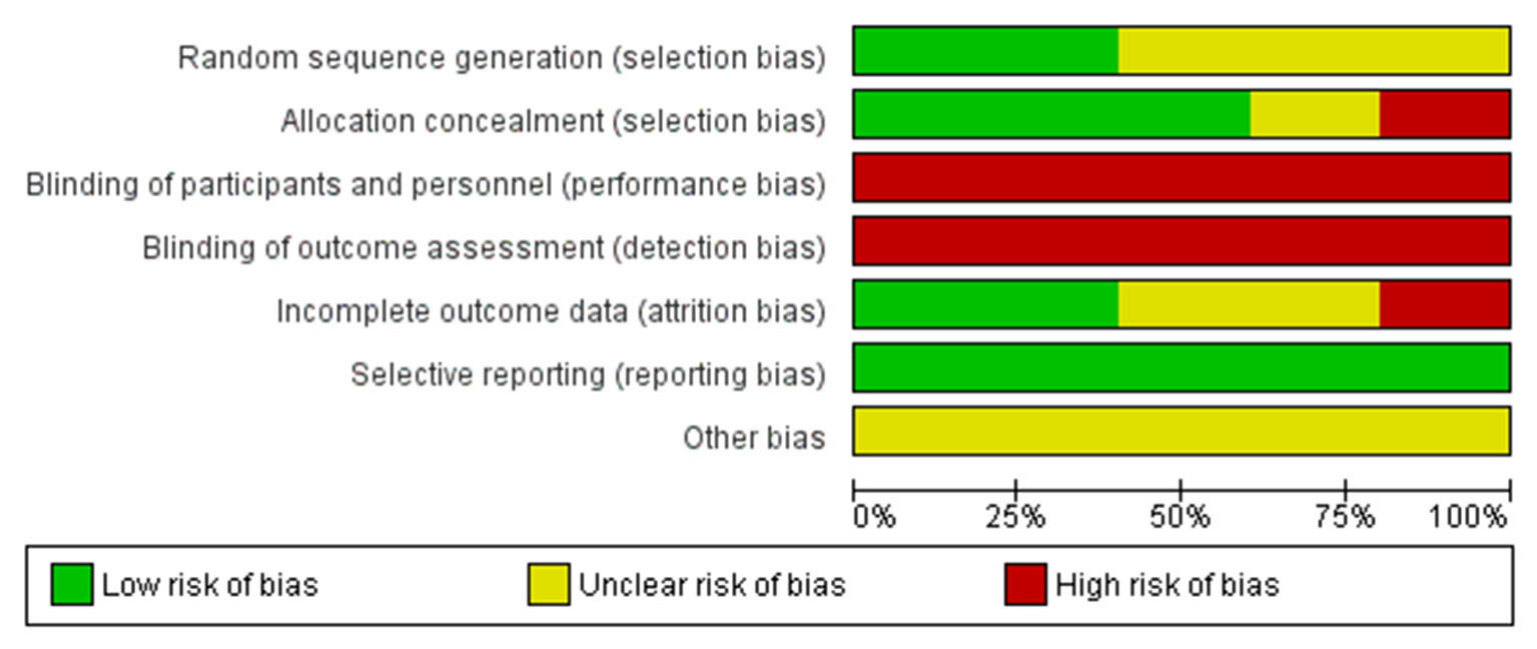
Bias assessment in RCTs.

A total of 100 items were identified through database research. Fourteen full-text papers were assessed for eligibility. Five of them did not report data concerning clinical outcomes or compliance of patients and were excluded (17, 19–21, 24). Five RCTs, two retrospectives, and two cohort studies were selected (Figure 1). In two RCTs and one non-RCT study, data were extracted from conference abstracts (25–27). All characteristics of the studies included are illustrated in Table 1 (fresh cycle) and Table 2 (frozen cycle). The risk of bias in non-RCTs is stated in Table 3, while the risk of bias in RCTs is illustrated in Figure 2.

**Table 1:** Characteristics of studies included (fresh cycle).

**Reference**	**Study type**	**Period of observation**	**Country**	**Cycle**	**Population**	**Intervention**	**Comparison**	**Results**
Baker et al. (28)	RCT	2009–2011	USA (8 centers)	IVF/ICSI	*n* = 782 Age 18-42 years BMI < 30 Kg/m^2^ FSH ≤ 15 IU/L Previous IVF < 3	*n* = 392 Subcutaneous Progesterone 25 mg/day	*n* = 390 Vaginal progesterone 200 mg/day	Comparable implantation rate, ongoing pregnancy rate, live birth rate. Comparable women satisfaction
Lockwood et al. (29)	RCT	2009–2010	Europe (13 centers)	IVF/ICSI	n = 683 Age 18-42 years BMI < 30 Kg/m^2^ FSH < 15 IU/L Previous IVF < 3	n = 339 Subcutaneous progesterone 25 mg/day	n = 344 Vaginal progesterone gel 90 mg (8%)/ day	Comparable ongoing pregnancy rate, live birth rate. Comparable women satisfaction
Mele et al. (30)	RCT	2017	Italy	IVF/ICSI	*n* = 130 Age < 37 years BMI 20–25 Kg/m^2^	*n* = 65 Subcutaneous progesterone 25 mg/day	*n* = 65 Intramuscular progesterone 33-50 mg/day	Comparable hCGβ pregnancy test. Higher PRL and Cortisol in women with intramuscular progesterone
Venturella et al. (26)	RCT (abstract)	2014–2016	Italy	IUI	*n* = 246 Age 18-38 years BMI 19-30 Kg/m^2^	*n* = 120 Subcutaneous progesterone 25 mg/day	*n* = 126 Vaginal progesterone gel 90 mg (8%)/ day	Comparable hCGβ pregnancy tes and clinical pregnancy rate Similar patients' satisfaction

*BMI, body mass index; FET, frozen embryo transfer; FSH, follicle stimulation hormone; IVF, in vitro fertilization; IUI, intrauterine insemination; n.a, not available; PRL, prolactin; RCT, randomized controlled trial*.

**Table 2:** Characteristics of studies included (frozen cycle).

**Reference**	**Study type**	**Period of observation**	**Country**	**Cycle**	**Population**	**Intervention**	**Comparison**	**Results**
Turkgeldi et al. (31)	Retrospective	2017–2017	Turkey	FET	*n* = 214 Age ≤ 43 years Previous IVF failed ≤ 3	*n* = 107 Subcutaneous progesterone 25 mg twice a day	*n* = 107 Vaginal progesterone gel 90 mg (8%) twice a day	Comparable βhCG pregnancy test, ongoing pregnancy rate, live birth rate and miscarriage rate
Gosalvez Vega et al. (27)	Prospective cross-over study (abstract)	2016	Spain	FET	*n* = 45	n = 45 Subcutaneous progesterone 25 mg/day	n = 45 Vaginal progesterone 800 mg/day	Subcutaneous progesterone was preferred to vaginal route
Ramos et al. (32)	Retrospective	2019	France	FET	*n* = 320 Age 18-42 years BMI 18-30 Kg/m^2^ No recurrent pregnancy loss	*n* = 160 serum progesterone ≤ 21.9 ng/ml (1-2 days before FET) subcutaneous progesterone 25 mg/day + vaginal progesterone 800 mg/day	*n* = 160 serum progesterone > 21.9 ng/ml (1-2 days before FET) subcutaneous progesterone 25 mg/day + vaginal progesterone 800 mg/day	Comparable implantation rate and clinical pregnancy rate Significantly lower miscarriage rate in women with serum progesterone above 21.9 ng/ml
Llacer et al. (25)	RCT (abstract)	n.a.	Spain	FET	*n* = 120 Oocyte donation recipients	n = 60 Subcutaneous progesterone 25 mg/day	n = 60 Vaginal progesterone 800 mg/day	Higher implantation rate, clinical pregnancy rate in women with vaginal progesterone Better compliance with subcutaneous progesterone
Venturella et al. (33)	Cohort study	2016	Italy	FET	*n* = 69 Age 18–43 years	*n* = 69 Subcutaneous progesterone 25 mg/day	*n* = 69 Vaginal progesterone	Better expectation and compliance with subcutaneous progesterone comparing with previous history with vaginal route

*BMI, body mass index; FET, frozen embryo transfer; FSH, follicle stimulating hormone; IVF, in vitro fertilization; IUI, intrauterine insemination; n.a., not available; PRL, prolactin; RCT, randomized controlled trial*.

**Table 3:** Newcastle-Ottawa scale for non RCTs.

	**Selection**	**Comparability**	**Esposure/Outcome**	**Total**
Gosalvez Vega et al. (27)	*	**	**	**5**
Turkgeldi et al. (31)	***	**	**	**7**
Ramos et al. (32)	**	**	**	**6**
Venturella et al. (33)	**	**	**	**6**

### Fresh Cycles

Two multicenter RCTs investigated the role of subcutaneous Pg in women undergoing fresh IVF cycles (28, 29). Lockwood et al. (29) designed a prospective, open-label, controlled, non-inferiority study involving 683 ART patients from 13 European centers randomized into two groups: subcutaneous Pg, 25 mg daily (*n* = 339); and vaginal Pg, 90 mg 8% gel daily (*n* = 344). Ongoing pregnancy rates at 10 weeks of treatment were 27.4% in women who underwent subcutaneous Pg and 30.5% in those who underwent vaginal gel, with a non-significant difference between the groups (−3.09%, 95% confidence interval [CI] 9.91–3.73). Consistently, live birth rate, miscarriage, and implantation rates were found to be similar between the groups. The overall incidence of non-serious adverse effects was also similar between the groups. While gastrointestinal and vaginal disorders (pruritus and discomfort) were more frequent with the use of vaginal gel, skin and subcutaneous reactions were more frequent using subcutaneous Pg. The second RTC was a prospective open-label non-inferiority trial conducted by Baker et al. (28) in 2014 and involved 800 women from eight clinics in the USA randomized to subcutaneous Pg (25 mg daily) vs. vaginal Pg (100 mg bid daily). The ongoing pregnancy rate was similar between the groups with a difference of −2.8% (41.6% subcutaneous Pg vs. 44.4% vaginal Pg). Comparable live birth rates, implantation rates, hCGβ, and clinical intrauterine pregnancies with fetal cardiac activity were observed. Both RCTs adopted similar eligibility criteria involving women with the retrieval of at least three oocytes aged between 18–42 years old (28, 29). Heterogenous OS was allowed in both trials in terms of pituitary suppression, gonadotropin regimen, and ovulation triggering. Furthermore, both RCTs used a −10% of non-inferiority margins for the lower limit of the two-sided 95% of CI. While Lockwood et al. (29) assumed an ongoing pregnancy rate of 30%, Baker et al. (28) considered an ongoing pregnancy rate of 43% in both groups.

A recent individual patient meta-analysis was carried out merging data from these two RCTs (34). The pooled risk difference in terms of ongoing pregnancy rates for subcutaneous Pg vs. vaginal Pg was −0.03 (95% CI −0.08–0.02). Consistently, the pooled risk difference regarding live birth rates was −0.02 (95% CI −0.07–0.03). In addition, the pooled odds ratio (OR) regarding OHSS risk was similar between the groups (OR 1.04, 95% CI 0.40–1.81).

A comparison between the intramuscular and subcutaneous routes was recently carried out by Mele et al. in 2020 (30). In detail, 130 women undergoing a first IVF cycle were randomized to intramuscular Pg (33 mg/day from ovum pick up and 50 mg/day from embryo transfer) and subcutaneous Pg (25 mg/day). The authors did not observe any difference in terms of the hCGβ pregnancy test between groups. Conversely, what was observed was significantly higher prolactin and cortisol levels measured seven days after ovum pick up in women who had undergone intramuscular Pg compared with those who had undergone a subcutaneous route. These data could suggest that the subcutaneous route might represent a solution to reduce stress and anxiety compared with the more painful intramuscular route.

### Frozen-Thawed Embryo Transfer (FET)

Frozen–thawed embryo transfer is widely used in ART. In contrast with a conventional fresh cycle, the transfer of embryos did not occur immediately after OS. This approach dramatically reduced the risk of OHSS and, at the same time, offered the possibility to transfer the embryos in a more physiological environment. Recent meta-analyses suggest that the transfer of cryopreserved embryos is associated with favorable perinatal outcomes (35–38).

Five studies explored the effects of subcutaneous Pg in a FET cycle (25, 27, 31–33). Specifically, a retrospective cohort study (31) involving 214 age-matched women compared 107 women who had undergone subcutaneous Pg (25 mg bid daily) with 107 women who had undergone vaginal Pg (90 mg 8% Pg vaginal gel bid daily). Only women <43-years-old and with <3 failed ART cycles were included (31). In both groups, only vitrified blastocysts reaching at least an expansion grade were transferred. Both groups showed comparable body mass index, antral follicle count, and the number of oocytes retrieved. Ongoing pregnancy rates (RR 1.11; 95% CI 0.78–1.56) and miscarriage rates (RR 1.08; 95% CI 0.76–1.55) were similar between groups.

Another retrospective trial explored the combination of both vaginal and Pg routes for LPS following FET (32). Two hundred thirteen women under 42-years-old, BMI between 18–30 kg/m^2^, and no history of recurrent miscarriage were included. Patients were stratified according to serum Pg measured 1–2 days before embryo transfer. LP was supported using vaginal formulation at a dose of 800 mg/day plus subcutaneous injections of Pg at a dose of 25 mg/day. Women with Pg levels above 21.9 ng/ml showed significantly lower miscarriage rates compared with those below these cut-off values. On the other hand, implantation and clinical pregnancy rates (gestational sac with heart activity) were similar, irrespective of Pg levels.

In a pilot randomized study, 120 oocyte recipients were randomized to receive subcutaneous Pg (25 mg daily) vs. vaginal Pg (200 mg bid daily) (25). The main aim of the investigators was to compare patients satisfaction through the administration of a questionnaire on comfort, hygiene, and sexual relations. Significantly better satisfaction was recorded using the subcutaneous route, although the ongoing pregnancy rate at 12 weeks of gestation was higher in women who had undergone a vaginal route (33.3 vs. 50%, *p* = 0.086) (25). In addition, significantly lower clinical pregnancy rates (36.8 vs. 59.3%, *p* = 0.022), implantation rates (32.5; 53.7% *p* = 0.017), and biochemical pregnancy per embryo transfer (26.3 vs. 5.6%, *p* = 0.004) were observed in women who had undergone a subcutaneous route compared with those who had undergone a vaginal route (25). In an open-label crossover study, Gosalvez-Vega et al. confirmed that subcutaneous Pg is better tolerated than the vaginal route in a FET cycle (27). Another study in the FET cycle was reported in an Italian prospective study involving 69 women with previous experience of vaginal Pg (33). In detail, patients were asked to complete three questionnaires (33). The first one regarded previous experience with a vaginal device, the second was at the time of transfer, and the third questionnaire was 8 days later evaluating their experience with subcutaneous progesterone. The authors reported better acceptance of the subcutaneous route compared with vaginal products.

### Intrauterine Insemination

Only one trial was developed to investigate the effect of subcutaneous Pg in women undergoing IUI (26). A total of 246 women were randomized to receive subcutaneous Pg (*n* = 126, 25 mg daily) or vaginal Pg gel (*n* = 120, 90 mg daily). Ongoing pregnancy rates per cycle (11.9 vs. 12.7 %) and hCGβ test (14.4 vs. 14%) were comparable between the groups, as was tolerability measured by a satisfactory score.

## Discussion

Subcutaneous Pg represents one of the most recent innovations proposed for LPS in IVF. This formulation was obtained by combining Pg with hydroxypropyl-b-cyclodextrin, which in turn increases the solubility of Pg (21). A preliminary RCT demonstrated that 25 mg/day or 50 mg/day of subcutaneous Pg can induce decidualization in 24 volunteers, whose ovarian function was suppressed with GnRH agonist and subsequently estrogenized using transdermal route (20). Sator et al. demonstrated that subcutaneous formulation was bioequivalent to intramuscular formulation in terms of the extent of exposure (19). In detail, the subcutaneous formulation is promptly absorbed and could achieve progesterone peak serum levels at an earlier time than the intramuscular route (19). In 24 donors undergoing OS and triggered with GnRHa, randomized to subcutaneous or intramuscular Pg, endometrial histology and endometrial receptivity array (ERA) transcriptomic expression showed non-significant differences in the secretory dating (24). Subcutaneous administration can be self-administered and is associated with lower injection site reactions, such as pain and irritation, compared with the intramuscular route (28). In addition, Mele et al. reported a significantly reduced level of stress-related hormones, such as Prolactin and Cortisol (30).

As an alternative to the vaginal route, subcutaneous Pg could be proposed to women who are reluctant to use vaginal medication for cultural or religious reasons. In addition, a vaginal route could be associated with vaginal irritation and discomfort (29), and the insertion of a vaginal device might be potentially associated with an increased risk of genital tract infection. Despite this belief, clinical data concerning genital (herpes virus, vaginal bacterial infection, vulvovaginal mycotic infection) and urinary tract infections seem to be similar when comparing vaginal and subcutaneous products (29). The potentially better compliance of subcutaneous vs. vaginal administration was investigated in several trials with mixed results. The most robust RCT trials (28, 29) conducted during fresh cycles did not demonstrate any relevant differences in terms of tolerability or adverse side-effects when comparing vaginal and subcutaneous administration. Conversely, two unpublished RCTs conducted in FET cycles reported higher satisfaction using the subcutaneous route. Similarly, Venturella et al. (33) observed better compliance with the subcutaneous route in a prospective study involving women with a previous experience of vaginal Pg; however, in FET cycles higher miscarriage and lower clinical pregnancy rates of the subcutaneous vs. the vaginal approach were reported in one RCT, involving oocytes donor recipients (25). This effect may be linked to the fact that the appropriate dosage in these populations is mainly unknown. So far, only one study compared intramuscular and subcutaneous Pg with no differences in terms of pregnancy rate between groups, but significantly lower prolactin and cortisol levels in the women who had received the subcutaneous route (30).

However, more evidence is necessary to better understand the application of subcutaneous Pg in ART. To the best of our knowledge, no RCT compared a subcutaneous Pg vs. oral dydrogesterone which could be a valuable approach for LPS in women undergoing ART (16, 39). In addition, so far no studies have been developed in women with a low prognosis to ART (40–46) or in women at risk of a hyper-response, such as those affected by PCOS (47–49). As yet, no cost-effective analysis has been conducted.

To conclude, a fair amount of evidence exists to support the hypothesis that in women with a good prognosis undergoing fresh IVF cycles, subcutaneous Pg is not inferior to vaginal products. In the FET cycle, data concerning efficacy are mixed with one RCT conducted in oocyte donor recipients which observed reduced clinical pregnancy rates and increased miscarriage rates in women undergoing a subcutaneous route. This data should be interpreted with caution considering that there is still too much uncertainty about the dosages to be used in women undergoing the FET cycle.

Data concerning the acceptance of a subcutaneous vs. vaginal route are encouraging despite the different scales and questionnaires used to test the acceptance of women among trials. Therefore, subcutaneous Pg could be proposed to women who are against vaginal administration and, in contrast with the intramuscular route, may be associated with better tolerability and reduced injection site reaction.

## Data Availability Statement

The original contributions presented in the study are included in the article/supplementary material, further inquiries can be directed to the corresponding author/s.

## Author Contributions

AC idealized the article and wrote the first draft. All authors participated in the literature research and paper editing. All authors listed have made an intellectual contribution to the work and approved the final version.

## Conflict of Interest

AC and CA decleare fees from Merck Serono Italia outside the submitted work. The remaining authors declare that the research was conducted in the absence of any commercial or financial relationships that could be construed as a potential conflict of interest.

## Publisher's Note

All claims expressed in this article are solely those of the authors and do not necessarily represent those of their affiliated organizations, or those of the publisher, the editors and the reviewers. Any product that may be evaluated in this article, or claim that may be made by its manufacturer, is not guaranteed or endorsed by the publisher.
